# The British Pharmacopœia

**Published:** 1898-06

**Authors:** 


**The British Pharmacopœia.** Pp. xxxii., 535. London: Spottiswoode & Co. 1898.

It is highly desirable that we should at the first possible opportunity notice the new edition of the *British Pharmacopœia* which has recently been issued. While its scientific standard is far in advance of its predecessors, especially in respect of pharmaceutical processes and botanical references, there yet remain some curious anomalies, due possibly to the prevailing of individual opinion of members forming the symposium by which the *Pharmacopœia* has been compiled. It is impossible to make here any attempt at a detailed analysis of the work, for that would occupy many pages. It is an absolute necessity for every practitioner within the next few months to make himself thoroughly acquainted with the numerous alterations which have been made, many of which are of importance.

It will be observed that the number of omissions of articles which were contained in the last edition largely exceeds in number the addition of novelties in the present issue: on looking over the list of these omissions and additions one is surprised at many of the useful preparations—such as ext. colchici acet.—being expunged, while other useless drugs—like hemidesmus—are retained: the additions consist mainly of new preparations, hardly any important drug being now made official for the first time, unless thyroid gland can be so considered.

By way of example of attempted improvements the tinctures may be taken as an instance; these have obviously been divided into two main groups in respect of the average dose. Of the more potent drugs the dose of the tincture is given as being from five to fifteen minims, the dose of the less powerful being given from half a drachm to a drachm. In order to reduce the tinctures thus into two ranks, it has been necessary to make the strengths of the tinctures of nux vomica and podophyllum twice as strong, and the ethereal tincture of lobelia one and a half times as strong, as in the old edition, while the tinct. chloroform. et morph. co. contains four times as much morphia as its predecessor. On the other hand, tincture of strophanthus is only half the strength. With these various alterations however, nothing like complete symmetry in dosage has been gained: tinct. conii is ordered in the small dose, tinct. hyoscyami in the large, and tinct. croci also in the small dose, while tinct. opii stands isolated midway between the two main classes. It is clear, therefore, that considerable alteration has been made in familiar preparations without corresponding advantage being obtained.

Omitting all reference to the various excellent appendices, which are of pharmaceutical rather than of medical interest, we cannot refrain from expressing unqualified admiration for the index. This must have cost great labour in preparation; besides a mere index, it is also a posological table and gives in addition the strengths of most preparations. This portion of the work is a veritable triumph, and will be the most useful part to those who consult the book.

We have received four small books dealing with the Pharmacopœia. These we hope to notice in a future number. They are issued respectively by Messrs. Evans, Gadd & Co.; Messrs. Ferris and Co.; the Pharmaceutical Journal Office; and Boot's Pure Drug Co. All seem likely to be useful. We understand that from July 1st, the local druggists will dispense the new preparations, unless other definite directions are given.

